# Finger joint motion generated by individual extrinsic muscles: A cadaveric study

**DOI:** 10.1186/1749-799X-3-27

**Published:** 2008-07-11

**Authors:** Ashish D Nimbarte, Rodrigo Kaz, Zong-Ming Li

**Affiliations:** 1 Hand Research Laboratory, Department of Orthopaedic Surgery, University of Pittsburgh, Pittsburgh, PA 15213, USA

## Abstract

**Background:**

Our understanding of finger functionality associated with the specific muscle is mostly based on the functional anatomy, and the exact motion effect associated with an individual muscle is still unknown. The purpose of this study was to examine phalangeal joints motion of the index finger generated by each extrinsic muscle.

**Methods:**

Ten (6 female and 4 male) fresh-frozen cadaveric hands (age 55.2 ± 5.6 years) were minimally dissected to establish baseball sutures at the musculotendinous junctions of the index finger extrinsic muscles. Each tendon was loaded to 10% of its force potential and the motion generated at the metacarpophalangeal (MCP), proximal interphalangeal (PIP), and distal interphalangeal (DIP) joints was simultaneously recorded using a marker-based motion capture system.

**Results:**

The flexor digitorum profundus (FDP) generated average flexion of 19.7, 41.8, and 29.4 degrees at the MCP, PIP, and DIP joints, respectively. The flexor digitorum superficialis (FDS) generated average flexion of 24.8 and 47.9 degrees at the MCP and PIP joints, respectively, and no motion at the DIP joints. The extensor digitorum communis (EDC) and extensor indicis proprius (EIP) generated average extension of 18.3, 15.2, 4.0 degrees and 15.4, 13.2, 3.7 degrees at the MCP, PIP and DIP joints, respectively. The FDP generated simultaneous motion at the PIP and DIP joints. However, the motion generated by the FDP and FDS, at the MCP joint lagged the motion generated at the PIP joint. The EDC and EIP generated simultaneous motion at the MCP and PIP joints.

**Conclusion:**

The results of this study provide novel insights into the kinematic role of individual extrinsic muscles.

## Background

The kinetics and the kinematics of the index finger have been studied extensively because of its vital role in numerous manual tasks. A series of grasping, pinching, and gripping tasks require coordinated flexion-extension motion by the index finger joints. A set of extrinsic and intrinsic muscles contribute collectively to achieve the precise force and motion essential for the dexterous finger maneuvers. Natural finger flexion and extension is achieved by linearly coupled motion among the metacarpophalangeal (MCP), proximal interphalangeal (PIP) and distal interphalangeal (DIP) joints [1,2]. A combined activation of the FDP and FDS using a biomechanical model demonstrated concurrent flexion at the MCP, PIP, and DIP joints [3]. The electrical stimulation of the FDP and FDS in live subjects also generated a comparable motion effect [3]. The in-vivo and in-vitro studies evaluating the fingertip force production [4,5] and grip strength [6] during maximal and submaximal exertions reported a high contribution from the extrinsic flexors and the intrinsic muscles, and a minimal contribution from the extrinsic extensors.

The local musculoskeletal architecture and its contribution to finger functionality were studied in several in vitro and biomechanical modeling investigations. Landsmeer presented a detailed discussion, based on his anatomical investigations, elaborating various scenarios of coordinated motion of the polyarticular finger joint system, with an emphasis on the structural constraints and the sequential actuation of multiple muscles [7]. Several biomechanical models have been constructed to estimate tendon excursions [8], moment arms [9,10], and muscle/tendon forces [11-13]. The anatomical distribution of the finger extensors was examined to understand their patterns of arrangement [14,15] and their structural variations [16]. The extensor mechanism of the fingers is complex and has been discussed in its anatomy [17] and function [18].

Though the index finger has been an object of extensive biomechanical investigation, there is a lack of knowledge concerning the kinematic role of individual muscles. The multiarticular nature of the extrinsic muscles, their potential to generate motion at multiple joints, and the associated redundancy in the actuation of the joints make it difficult to comprehend the functionality of individual muscles. Therefore, the purpose of this study was to investigate the index finger joint motion generated by individual extrinsic muscles. Cadaveric hand specimens were used to simulate precisely the force exertion by individual extrinsic muscles. The flexion/extension motion generated at the MCP, PIP, and DIP joints by the individual extrinsic muscles was evaluated.

## Methods

### 2.1 Specimen preparation

Ten (6 female and 4 males) fresh-frozen human cadaveric hand specimens (age 55.2 ± 5.6 years) were used in this study. The specimens were amputated at the mid-humerus and were free from apparent musculoskeletal disorders. After thawing overnight at room temperature, the specimens were minimally dissected to expose the musculotendinous junctions of the extrinsic muscles: the flexor digitorum profundus (FDP), the flexor digitorum superficialis (FDS), the extensor digitorum communis (EDC), and the extensor indicis proprius (EIP). Baseball sutures were made at the musculotendinous junctions for the purpose of tendon loading.

### 2.2 Specimen mounting

**Figure 1:**
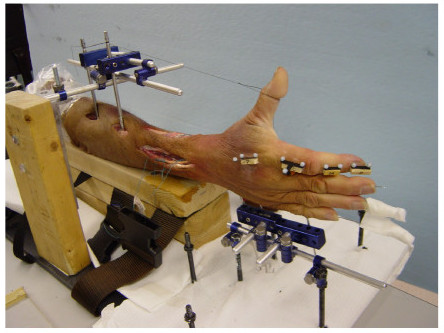
A cadaveric hand mounted on a custom fixation device for tendon loading and motion recording.

Each specimen was mounted on an experimental table using a custom-built fixation apparatus (Figure 1). The forearm was stabilized in neutral pronation/supination with the elbow fixed in 90 degrees of flexion. Two schanz screws (5 mm diameter) were drilled vertically into the proximal part of the radius by using an open approach to facilitate forearm stabilization. Three additional schanz screws (2.5 mm diameter) were drilled into the ulnar aspect of the hand; two were drilled into the metacarpal bone of little and ring finger, and one was passed through the phalanx of little, ring, and middle fingers, respectively. Subsequent to forearm stabilization, the implanted schanz screws were clamped, mounting the wrist in the neutral position. The MCP, PIP, and DIP joints of the little, ring, and middle fingers were locked into full extension using transarticular 1.6-mm Kirschner wires, which were placed in a retrograde manner through the tip of the fingers across the phalangeal joints. The thumb was kept fully extended, parallel to the palmer plane of hand.

### 2.3 Data collection

Motion data were recorded using a motion analysis system (Vicon 460, Oxford, UK). A set of ten reflective markers (5 mm in diameter) was placed on the dorsal surface of the index finger; 2 markers were placed on the distal phalanx, and 2 markers were placed on the middle phalanx. Two T-shaped plates with 3 markers on each plate were placed on the proximal phalanx and the second metacarpal, respectively (Figure 1). During the data collection, each tendon was pulled manually using a force transducer (Nano 17, ATI Industrial Automation, Apex, NC). One end of the transducer was connected to a suture at the musculotendinous junction and the other end of the transducer was clamped on a rectangular wooden handle (4 cm × 4 cm × 8 cm) to facilitate manual gripping and pulling. Each tendon was loaded to 10% of its force potential, i.e., 13.2 N, 9.8 N, 4.9 N, and 4.9 N for the FDP, FDS, EDC, and EIP, respectively [19]. The forces were applied, from 0 to the target in 10 seconds, following a ramp displayed on the screen by a custom LabVIEW program. At the beginning of each trial the line of force application via the pulling string was visually aligned with the muscle/tendon line. Three trials of pulling were collected for each muscle tendon. Prior to the actual pulling, the index finger was preconditioned to standardize the positions of the joints. During the preconditioning, the finger was manually moved for 15 seconds through its comfortable motion territory and then placed in a position with minimal resistance to motion. Same experimenter performed the preconditioning of all the specimens for the purpose of consistency and accuracy.

### 2.3 Data analyses

The flexion angles for the MCP, PIP, and DIP joints were determined by performing vector calculations assuming that the joint motion occurred in the flexion/extension plane. The resting joint position after the preconditioning without a pulling force was defined as the starting position. The joint range of motion was determined by subtracting the joint flexion angle at the target force (10% of the maximum force) from the starting position angle. Regression analyses were performed to examine the motion coordination among the joints.

## Results

**Figure 2:**
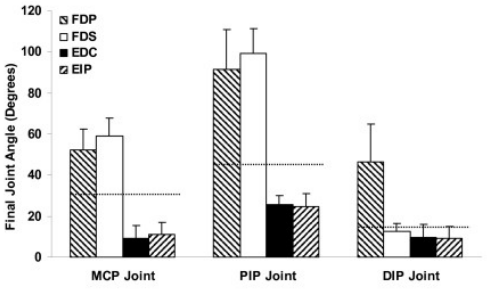
**Final joint angles (degrees) generated at the MCP, PIP, and DIP joints by the individual extrinsic muscles when loaded to 10% of their respective maximum force potentials.** The dotted lines indicate the starting joint positions.

**Figure 3:**
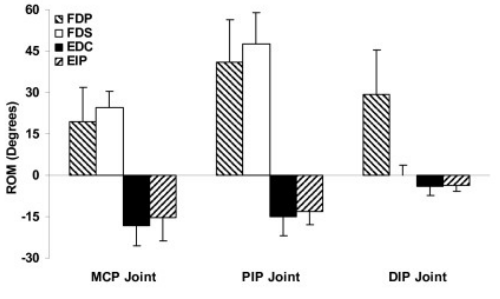
**The ranges of motion (degrees) generated at the MCP, PIP, and DIP joints by the individual extrinsic muscles when loaded to 10% of their respective maximum force potentials.** Positive values indicate flexion and negative values indicate extension. The range of motion values are with respect to the starting position.

**Figure 4:**
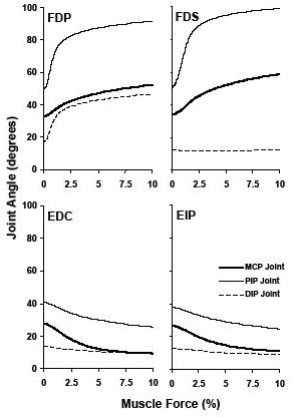
**Angular trajectories generated by the individual extrinsic muscles.** The muscle forces are expressed as the percentages of their maximum force potentials. The force-angle curves were averaged across the 10 specimens.

The average starting positions of MCP, PIP, and DIP joints were at flexion angles of 30.3 (± 9.0), 45.1 (± 10.2), and 14.2 (± 5.9) degrees, respectively. The FDP tendon loaded to 10% force produced the final joint positions of 52.4 (± 11.9), 91.5 (± 17.0), and 46.6 (± 17.8) degrees at the MCP, PIP, and DIP joints, respectively (Figure 2), corresponding to respective ranges of motions of 19.7 (± 12.4), 41.8 (± 15.0), and 29.4 (± 17.0) degrees (Figure 3). The range of motion ratios (MCP:PIP:DIP) were 1:2.1:1.5. The motion trajectories at the MCP joint showed a gradual ascending pattern in the 0–10% force range (Figure 4); in contrast, the motion trajectories at the PIP and DIP joints showed a rapid rise in the small tendon force range (<3%), leveling off as the tendon force continued to increase (Figure 4). The PIP joint flexion positions at the 2.5%, 5%, and 7.5% forces were 82.4 (± 19.5), 87.2 (± 19.2), and 89.7 (± 18.1) degrees, respectively, representing 78.2%, 89.8%, and 95.8% of the total range of motion, respectively. Similarly, the DIP joint reached at flexion positions of 38.9 (± 17.9), 42.9 (± 18.1), and 45.1 (± 17.9) degrees at the 2.5%, 5%, and 7.5% forces, respectively, representing 73.9%, 87.5%, and 94.8%, of the total range of motion, respectively. The flexion movements at the PIP and DIP joints occurred simultaneously with a correlation coefficient of 0.979 (± 0.028). The slope of the linear regression of DIP joint angle as a function of PIP joint angle was 0.692 (± 0.358), meaning that one degree of PIP joint flexion was linearly coupled with approximately 0.7 degrees of DIP joint flexion.

The loading of the FDS tendon to 10% force produced final joint positions of 58.9 (± 9.5) degrees at the MCP joint and 99.1 (± 11.0) degrees at the PIP joint (Figure 2), representing ranges of motion of 24.8 (± 5.6) and 47.9 (± 11.6) degrees, respectively (Figure 3). However, there was no range of motion (0.0 ± 3.6 degrees) generated at the DIP joint. The range of motion ratio (MCP:PIP) was 1:1.9. Similar to FDP tendon loading, the motion trajectories at the MCP joint showed a relatively gradual ascending pattern in the 0–10% force range, and the motion trajectory at the PIP joint was asymptotic (Figure 4). At the 2.5%, 5.0%, and 7.5% forces, the PIP joint flexion position reached at 88.5 (± 12.6), 94.8 (± 12.1), and 97.4 (± 11.5) degrees, respectively, representing 77.9%, 91.1%, and 96.5% of the total range of motion, respectively.

The loading of EDC tendon to 10% force produced final joint positions of 9.4 (± 3.8), 25.5 (± 4.1) and 9.7 (± 6.4) degrees at the MCP, PIP, and DIP joints, respectively (Figure 2), corresponding to the respective ranges of motion of 18.3 (± 7.4), 15.2 (± 6.6) and 4.0 (± 3.2) degrees (Figure 3). The loading of the EIP tendon to 10% force produced a final joint position of 11.2 (± 2.7), 24.6 (± 6.7), and 9.2 (± 6.1) degrees at the MCP, PIP, and DIP joints, respectively (Figure 2), corresponding to the respective ranges of motion of 15.4 (± 9.0), 13.2 (± 4.5), and 3.7 (± 2.5). The range of motion ratios (MCP:PIP:DIP) were 1:0.8:0.2 for both the EDC and EIP. The patterns of motion trajectories at the individual phalangeal joints generated by the EDC and EIP were also similar (Figure 4). The loading of the EDC or EIP generated simultaneous extensions at the MCP and PIP joints. The correlation coefficients between the MCP and PIP joint movements were 0.951 (± 0.092) for the EDC and 0.973 (± 0.037) for the EIP. Linear regression analyses by PIP joint angle as a function of MCP joint angle showed that the average slopes of linear regression line were 0.77 for the EDC and 0.75 for the EIP.

## Discussion

In this study, the joint motion of the index finger induced by each extrinsic muscle was examined using a marker-based motion capture system together with a tendon force monitoring sensor. The protocol used in this study, i.e., gradual pulling of the musculotendinous junction of the index finger extrinsic muscles, simulated the contracting muscle, allowing us to quantify the resulting motion trajectories at the phalangeal joints.

The results of this study show that the individual extrinsic muscles generated motion at multiple index finger phalangeal joints. Whereas the FDP generated motion at all the phalangeal joints, the FDS, as expected, did not generate motion at the DIP joint. The EIP and EDC generated similar phalangeal joint motion. Using biomechanical model, Kamper et al. found that the combined actuation of FDP and FDS generated ranges of motion of 43, 75, and 63 degrees at the MCP, PIP, and DIP joints, respectively [3]. Becker and Thakor obtained range of motion data from 15 human subjects: 70.8, 103.8, and 61.2 degrees for the MCP, PIP, and DIP joints, respectively [20]. However, the motion data in the current study are not directly comparable with the motion data involving activation of multiple muscles.

To a great extent, the anatomical arrangement of the tendons explains observed joint motion. The extrinsic flexors originate in the forearm, cross over multiple joints, and insert into either the middle or the distal phalanx. The FDP spans the MCP, PIP, and DIP joints, inserting at the base of distal phalanx, and the FDS spans the MCP and PIP joint, inserting at the middle phalanx. Therefore, the FDP generated motion at the MCP, PIP and DIP joints, while the FDS generated motion at the MCP and PIP joints. The extensor tendons pass over the MCP joint and trifurcate into medial, central, and lateral slips at the PIP joint. The central slip inserts into the base of middle phalanx, while the medial and lateral slips pass on either side of the PIP joint, inserting into the distal phalanx [21]. The extensors mainly generate motion at the MCP and PIP joints as observed in this study. The encircling series of fibers (sagittal band) that are connected to the extensors enhance the MCP joint motion [18]. The migration of the medial and the lateral slips toward each other generate DIP joint extension. The tighter the proximal pull on the extensors, the more closely the slips migrate towards each other producing additional extension of the DIP joint [18]. The force applied in this study (i.e., 10% of the maximum force) might not have relocated the slips enough, producing relatively small extension motion (<5 degrees) at the DIP joint. It is also known that DIP extension is mainly achieved by the activation of the intrinsic muscles through the extensor mechanisms [16,22].

The motion generated at the phalangeal joints showed strong interjoint coordination. The EDC and EIP tendons generated simultaneous, linearly coupled motion at the MCP and PIP joints. Although the FDP tendon also generated simultaneous, linearly coupled motion at the PIP and DIP joints, the FDP and FDS tendons generated a distinct inter-joint coordination pattern at the MCP and PIP joints. Within the 0–3% force range, the motion generated at the PIP joint (34.1 ± 17.1 degrees by FDP; 39.3 ± 12.3 degrees by FDS) was almost three times the motion generated at the MCP joints (10.7 ± 7.9 degrees by FDP; 13.2 ± 4.1 degrees by FDS). The motion generated (within 0–3% force range) at the PIP joint was approximately 80% of its total range of motion. However, the motion generated at the MCP joint was approximately 50% of its total range of motion. The loading of flexors between 3–10% generated comparable ranges of motion at the MCP (8.9 ± 4.8 degrees by FDP; 11.6 ± 2.5 degrees by FDS) and PIP (7.7 ± 3.5 degrees by FDP; 8.6 ± 3.3 degrees by FDS) joints. Thus, in the 0–3% force range, the PIP joint flexion leads the MCP joint flexion. The joint impedance could be a possible reason for this observation. The passive joint characteristics such as the number of muscles crossing the joint could govern its impedance. The MCP joint has more muscles crossing it than the PIP joint, providing higher impedance and thus less motion at the MCP joint. The joint impedance increases as it flexes. After a certain level of flexion, the impedance of the PIP joint becomes equivalent to the MCP joint causing similar motion at both joints. In the current study this point of matched impedance was observed after the 3% force.

Based on the motion data determined in this study, it is possible to speculate some role of the intrinsic muscles in phalangeal joint motion. We comprehend three possible movement manifestations of the intrinsic muscles. First, the intrinsic muscles assist in the flexion of the MCP joint. An extrinsic flexor with a 10% force generated almost full flexion at the PIP joint, but only a sub maximal joint flexion at the MCP joint. Kamper et al. [3] stated that the activation of the intrinsic muscles may be necessary to produce MCP flexion beyond 60 degrees. Darling et al. [2] observed activation in the intrinsic muscles during finger flexion, especially during fast flexion movements. Hence, activation of intrinsic muscles is necessary to generate extreme and fast flexion at the MCP joint. Second, the intrinsic muscles facilitate the finger movements that require simultaneous flexion and extension at the MCP and IP joints, respectively. Darling et al. observed simultaneous movements of the finger joints during various daily living grasp and release tasks [2]. The pulling of extrinsic muscles generated either concurrent flexion or extension at the MCP and the IP joints, i.e. the joints were rotated in one direction only. Landsmeer and Long stated that lumbricals and interosseous were involve in the motion generating MCP flexion with simultaneous extension of the IP joints [22]. Kamper et al. speculated that intrinsic muscles may assist MCP flexion indirectly by increasing the resistance to the IP flexion [3]. Thus, flexing MCP joints, while retaining the IP joints at relatively extended positions, may require contribution from the intrinsic muscle. Third, the intrinsic muscles are responsible for the abduction-adduction of the MCP joint. Though abduction-adduction motion was not quantified in this study, during the data collection negligible abduction-adduction motion was observed at the MCP joint due to the actuation of the individual extrinsic muscles. It follows that the abduction-adduction motion available to the MCP is generated by the intrinsic muscles. The intrinsic muscles are oriented on the radial and ulnar side of the MCP joint, which facilitates both abduction and adduction of the MCP joint. Future studies delineating the motion contribution of the intrinsic muscles would be useful in drawing concrete conclusions about their role in phalangeal joint motion. However, such studies will be faced with the challenges of not disturbing the local anatomy and retaining the intact kinematics.

We acknowledge several limitations of this study. First, the mechanical properties of the cadaveric tissues may be different from live tissues. The relationship between the muscle force and joint movements is dependent upon the mechanical characteristics of the joint. The intrinsic muscles, articular cartilage, ligaments, joint capsule, synovial fluid, and opposing bone surface are determinants of the mechanical characteristics of a joint. Moreover, the cadaveric specimens were mostly obtained from the elderly subjects, whose stiffness might be higher than those of young subjects. These discrepancies could affect the joint characteristics (e.g., impedance), limiting the generalization of our results to live hands and a diverse age population. Future studies evaluating changes (in vivo vs. in vitro) in the joint characteristics could be useful in extending the results of this study to live hands. In vivo studies using electrical stimulation could also be performed to validate the kinematic role of the individual extrinsic muscles. Second, the tendons were loaded to only 10% of their maximum force potentials. This loading force was judged reasonable because phalangeal motion requires sub-maximal activation of the muscles, and the purpose of this study was to examine the motion (not the strength) generated by the individual tendons. Nevertheless, most of the joint motion trajectories tended to level off at the higher end of the force application. Third, a moderate level of variability was observed in the starting joint positions, which could be due to the subjective preconditioning procedure and the accidental tension prior to muscle loading. The latter scenario is more probable because the starting joint position was found to be shifted in the direction of the movement, i.e., having a more flexed starting position for the flexors and more extended starting position for the extensors. This experimental artifact might have decreased the ranges of motion by 3–5 degrees. Finally, to standardize hand and wrist mounting positions, the phalangeal joints of the middle, ring, and little fingers were locked into full extension, which might have restricted the flexion mobility of the finger motion, especially at the MCP joint.

Despite of these limitations, the results of this study provide novel insights into the functional manifestation of the index finger joints by individual extrinsic muscles.

The knowledge of the kinematic role of individual muscles could be used for designing neuromuscular electrical simulation protocols [23], reconstructive surgeries [24], and planning of diagnosis and treatment modalities of the finger joint problems.

## Conclusion

The quantitative motion generated by the individual extrinsic flexors and extensors at the index finger phalangeal joints is documented in this study. Distinct motion trajectories were generated at the phalangeal joints by the individual extrinsic muscles. The FDP generated motion at the MCP, PIP, and DIP joints, while the FDS generated motion only at the MCP and PIP joints. The PIP joint was flexed the most, followed by the MCP and DIP joints. The FDP generated simultaneous motion between the PIP and DIP joints and the motion generated at the PIP joint lead the motion at the MCP joint for the flexors. The extensors generated comparable and simultaneous motion at the MCP and PIP joints; relatively small motion was generated at the DIP joint.

## Competing interests

The authors declare that they have no competing interests.

## Authors' contributions

ADN designed the study, collected and analyzed the data and drafted the manuscript. RZ assisted with the preparation of cadaveric specimens and participated in drafting the manuscript. ZML conceived the project and helped with the experimental design, data analyses and manuscript writing. All authors read and approved the final manuscript.
